# Advanced tsunami detection and forecasting by radar on unconventional airborne observing platforms

**DOI:** 10.1038/s41598-020-59239-1

**Published:** 2020-02-12

**Authors:** Iyan E. Mulia, Tomoyuki Hirobe, Daisuke Inazu, Takahiro Endoh, Yoshihiro Niwa, Aditya Riadi Gusman, Hidee Tatehata, Takuji Waseda, Toshiyuki Hibiya

**Affiliations:** 1 UTokyo Ocean Alliance, The University of Tokyo, Tokyo, Japan; 2 Earthquake Research Institute, The University of Tokyo, Tokyo, Japan; 3 Japan Weather Association, Tokyo, Japan; 4 Department of Marine Resources and Energy, Tokyo University of Marine Science and Technology, Tokyo, Japan; 5 Research Institute for Applied Mechanics, Kyushu University, Fukuoka, Japan; 6 Center for Ocean Literacy and Education, The University of Tokyo, Tokyo, Japan; 7 GNS Science, Lower Hutt, New Zealand; 8 Graduate School of Frontier Sciences, The University of Tokyo, Tokyo, Japan; 9 Graduate School of Science, The University of Tokyo, Chiba, Japan

**Keywords:** Natural hazards, Physical oceanography, Seismology

## Abstract

Sustaining an accurate, timely, and global tsunami forecast system remains a challenge for scientific communities. To this end, various viable geophysical monitoring devices have been deployed. However, it is difficult to implement new observation networks in other regions and maintaining the existing systems is costly. This study proposes a new and complementary approach to monitoring the tsunami using existing moving platforms. The proposed system consists of a radar altimeter, Global Navigation Satellite Systems receiver, and an adequate communication link on airborne platforms such as commercial airplanes, drones, or dedicated high-speed aircraft, and a data assimilation module with a deterministic model. We demonstrated, through twin-data experiment, the feasibility of the proposed system in forecasting tsunami at the Nankai Trough of Japan. Our results demonstrated the potential of an airborne tsunami observation as a viable future technology through proxy observations and rigorous numerical experiments. The wide coverage of the tsunamigenic regions without a new observation network is an advantage while various regulatory constraints need to be overcome. This study offered a novel perspective on the developments in tsunami detection and forecasting technology. Such multi-purpose observation using existing platforms provides a promising and practical solution in establishing sustainable observational networks.

## Introduction

Early detection of tsunamis in the open ocean is one of the crucial factors for tsunami forecasting, and hence tsunami observing systems have been mainly deployed in the deep waters. There are several established systems that are currently operating: the Deep-ocean Assessment and Reporting of Tsunamis (DART)^1^, the Japanese cabled ocean bottom pressure systems including the Dense Oceanfloor Network System for Earthquakes and Tsunamis (DONET)^2^ and S-Net^3^, and the Canadian North–East Pacific Time-series Underwater Networked Experiments (NEPTUNE-Canada)^4^. In addition to the bottom pressure systems, some surface measurements based on Global Positioning System (GPS) buoys are also available along the coasts of Japan^5^. These existing systems have detected numerous tsunamis and their viabilities are proven. However, these devices are attached to conventional platforms at fixed locations and their sustainability is a concern. One alternative is the utilization of satellite altimetry data to detect a tsunami^6,7^, but the sparseness limits their capability. A more advanced space-borne observation using a global navigation satellite system reflectometry can be a prospective offshore tsunami detector^8^. However, satellites are costly and less expensive system utilizing existing platforms is desired. Recently, several studies suggested the use of spontaneous/moving observations by a ship height positioning to detect tsunamis^9,10^. What is intriguing is that such a system offers a unique and most likely wider coverage than the conventional observing systems.

An essential component of a fully functioning tsunami forecasting system is the data assimilation. The tsunami waveform inversion, which is the most common tsunami forecasting method, utilizes the pre-calculated Green’s functions to estimate the initial values of the numerical model. Tsushima *et al*.^11^ developed the so-called tFISH/RAPiD (tsunami Forecasting based on Inversion for initial sea-Surface Height/Real-time Automatic detection method for Permanent Displacement), which has been integrated to the Japan Meteorological Agency tsunami forecasting system. Another application of the tsunami waveform inversion is the Short-term Inundation Forecast for Tsunamis (SIFT) implemented at the National Oceanic and Atmospheric Administration Tsunami Warning Centers^12^. More recently, a sequential tsunami data assimilation method based on optimal interpolation has been proposed^13^ and was applied to several events^14–16^. The variant of this scheme, which is suitable for spontaneous observations such as sea surface measurement by ship height records, was developed combining the Green’s function-based initialization^17^. However, considerable improvement is still needed to efficiently cope with high-speed moving platforms such as airborne observations.

Our previous study^18^ demonstrated the successful observation of sea surface heights (SSH) across the Kuroshio current as a proxy data with a sufficient level of error for the detection of large tsunamis. Accordingly, here we propose a new approach to monitoring the tsunami based on the same instrumentation of radar altimeter and global navigation satellite systems (GNSS) receiver installed on either commercial airplanes (CAs), drones, or dedicated high-speed aircrafts (HAs). The procedure is, in principle, similar to that using space-borne observations by satellite altimetry. But, the average relative speed of airplanes is much slower than satellites, enabling us to observe the tsunami for a longer duration to be adequately assimilated into the numerical model. We develop an efficient data assimilation procedure for moving observations, extending the optimal interpolation scheme developed for fixed observation points^13–17^. For fast-moving observation points, the error covariance matrix needs to be updated at each assimilation sequence which requires considerable computational time. To minimize the computational time, the dimension was substantially reduced by means of low-pass filtering the spectrum of the error covariance matrix.

## Results

**Figure 1:**
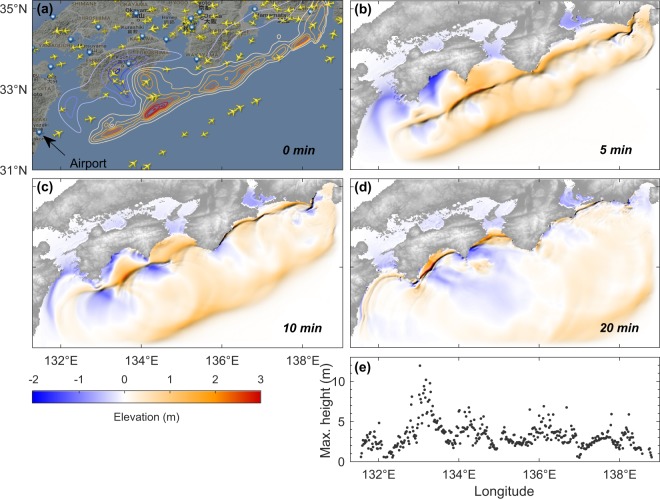
(**a**) Initial CAs distribution based on flight tracking data on 15 November 2016, 05:00 UTC (www.flightradar24.com). Contour lines indicate sea surface displacements of a hypothetical tsunami source generated by an earthquake of M8.7 (obtained from the CDMC report^19^), or the true state at t = 0 min. (**b**–**d**) The true state of tsunami elevations at 5, 10, and 20 min. (**e**) Maximum coastal tsunami heights along the longitudinal axis associated with the source obtained after 60-min simulation. Image in (**a**) is obtained from https://www.flightradar24.com/ (data: Google Maps, 2016, flightradar24) overlaid with contour generated using MATLAB 2016b. Maps in (**b–d**) are produced by authors based on GEBCO_08 Grid data (https://www.gebco.net/), using MATLAB 2016b.

**Figure 2:**
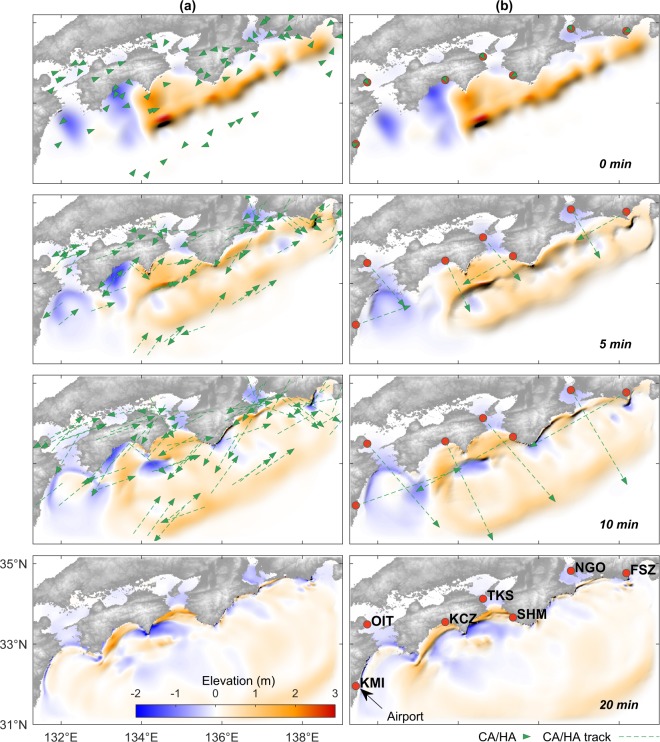
Assimilated tsunami elevations with 10-min assimilation period and 60-min simulation time. The initial condition (at t = 0) is obtained from the inversion analysis. (**a**) Using 65 CAs with a uniform speed of 200 m/s. (**b**) Using 7 HAs with a uniform speed of 500 m/s. Red dots indicate the selected airports: Miyazaki (KMI), Oita (OIT), Kochi (KCZ), Tokushima (TKS), Shirahama (SHM), Chubu (NGO), and Shizuoka (FSZ). Maps in (**a**,**b**) are produced by authors based on GEBCO_08 Grid data (https://www.gebco.net/), using MATLAB 2016b.

To assess the proposed approach, we utilize the result of a numerical simulation of the potential future Nankai Trough tsunami generated by a hypothetical M8.7 earthquake obtained from the Central Disaster Management Council (CDMC), Japan^19^ (Fig. 1a–d). The CDMC source represents a plausible key feature of future events based on the seismotectonic settings of the region and it had been widely used in the corresponding studies e.g.^10,17^. Additionally, we also store the associated maximum coastal tsunami heights for validation purposes (Fig. 1e). For a concise presentation, the figures show only 20-min simulation results, albeit the actual simulation time is 60 min. We also store the spatiotemporal variations of simulated tsunami elevations along assumed airplane tracks and add real noise obtained from our observations^18^ to imitate the actually observed tsunami in the real event. Assuming that the tsunami signal together with flight tracking data is available in real-time, we can then perform the proposed tsunami data assimilation to make rapid tsunami forecasts. An initial guess of the background state is roughly estimated using the tsunami source by an inversion analysis from existing tsunami observing systems in the region^17^ (t = 0 in Fig. 2a,b). Details about the experimental setup are described in Supplementary #1 and illustrated in Fig. S1. We use statistical measures $K$ and $\kappa$ based on Aida^20^ with the suggested criteria by Shuto^21^ to validate our results (see Supplementary #1).

### Commercial airplanes

Real-time flight tracking data together with other flight information for CAs are presently available using a surveillance technique based on an automatic dependent surveillance-broadcast (ADS-B)^22,23^. There are several global flight tracking services derived from the ADS-B that can be accessed online, e.g., FlightAware, Flightradar24, and Plane Finder. In this study, we use a snapshot of CAs distribution on 15 November 2016 (05:00 UTC) obtained from www.flightradar24.com (Fig. 1a). The virtually observed tsunami are generated under the assumption that the earthquake-induced tsunami occurs at time t = 0 when the initial airplane distribution is extracted from the Flightradar24. Then, the flight tracks are extended along straight lines from the initial heading directions at a uniform speed of 200 m/s (the average speed of passenger airplanes is ranging from ~200–250 m/s).

**Figure 3:**
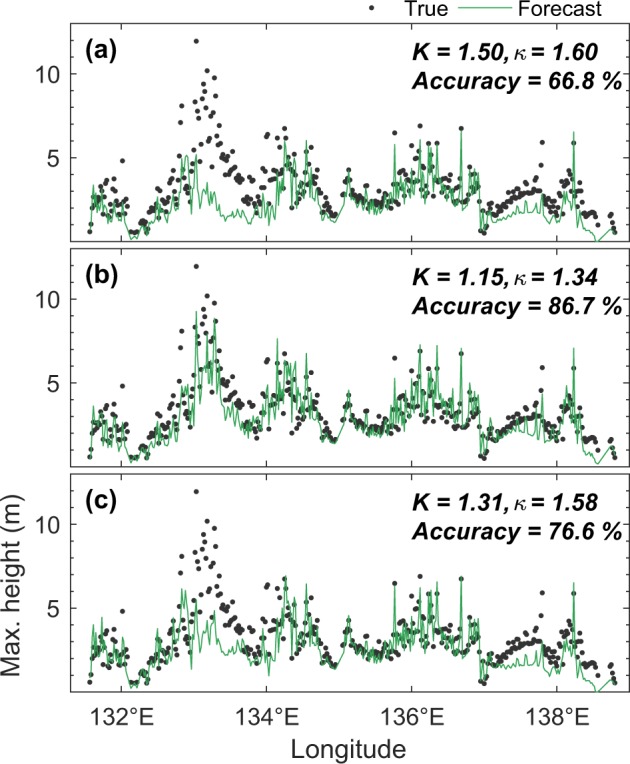
Comparison of true and forecasted maximum coastal tsunami heights obtained after 60-min simulation. Formulation of the statistical measures of $K$, $\kappa$, and accuracy are presented in Supplementary #1. Note that for a concise presentation, *K* and κ values are rounded to two decimal places, but the accuracy is calculated based on the original values without rounding. (**a**) Without assimilation. (**b**) Assimilation result using 65 CAs associated with Fig. 2a. (**c**) Assimilation result using 7 HAs associated with Fig. 2b.

Figure 2a illustrates the data assimilation process using 65 CAs. Without assimilating the SSH observed by the CAs, the forecast of maximum coastal tsunami heights results in $K$ = 1.50, and $\kappa$ = 1.60, and accuracy of 66.8% (Fig. 3a), which can also be considered as the forecast performance by the nonoptimal source estimate. The shortage of this forecast is mainly attributed to the limited inversion period to account for the near-field source being considered (we use a 10-min window so that it is consistent with the assimilation period) and the lack of spatial coverage of observation points used to infer the tsunami source^17^. With the assimilation of SSH observed by the CAs, the tsunami wavefield originating from the less accurate initial tsunami source estimation is gradually corrected, leading to a considerable improvement of forecast performance ($K$ = 1.15, $\kappa$ = 1.34, accuracy = 86.7%) (Fig. 3b). Given the limited time and data, especially in near-field events, the accuracy of real-time tsunami source estimates is usually poor^24,25^. Our experiment shows that within the same time frame, additional data can efficiently be incorporated in the forward modeling stage to improve the forecasts.

In the real case, however, the result is dependent upon the availability of airplanes. In Fig. S3 (Supplementary #2), we plot the CA distribution around the study area for six consecutive days at the same hour (05:00 UTC) including the one used in Fig. 2a. During the day, the distribution of CAs (on average of 50 to 60 airplanes) is sufficient to obtain a satisfactory result as indicated by the previous experiment. However, one should expect fewer airplanes in the night time. Figure S4 shows variations of CA distribution throughout Japan on the same day at 4-hour intervals. We use more recent data from the flightradar24 on 25 July 2018. To assess the forecast performance when a limited number of airplanes exist in the study area, we use the distribution of 8 CAs at 16:00 (UTC). The results are plotted in Fig. S5, which show that the forecast accuracy is below the satisfactory criteria^21^, but still better than the initial forecast depicted in Fig. 3a.

### High-speed aircrafts

**Figure 4:**
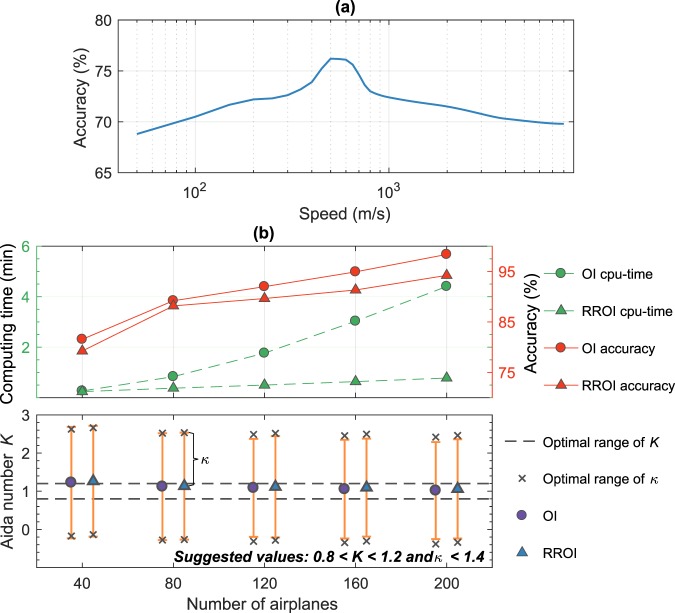
(**a**) Variation of forecast accuracies by 7 HAs with different speeds. (**b**) Computing times and accuracies by different number of CAs.

In this experiment, we assume that specifically dedicated HAs for tsunami observations are available at any time each at seven airports around the study area (see Fig. 2b), and are scrambled immediately after the earthquake toward offshore. The HA can be either common military aircraft or high-speed unmanned aerial vehicles (UAV)^26,27^. The use of HA can provide flexibility in achieving the desired speed and route. Therefore, the optimal speed of 500 m/s suggested by the experimental result shown in Fig. 4a, can easily be implemented here. The forecast performance by assimilating SSH observed using 7 HAs (Fig. 2b) yields an accuracy of 76.6%, $K$ = 1.31, $\kappa$ = 1.58 (see Fig. 3c), which is substantially smaller than the case of 65 CAs that yielded 86.7%. However, this is comparable to the 8 CA case shown in Fig. S5, and highlights the advantage of HA. While the 8 CAs immediately record the tsunami at their offshore initial locations, the 7 HAs require additional time before starting the detection. Therefore, a faster speed is necessary not only to ensure sufficient spatial coverage controlling the forecast accuracy but also to compensate for the unfavorable initial observation locations. It is evident from this experiment that with the optimal speed and a certain initial distribution, HAs can still produce a reasonable level of accuracy with a much less number of observations compared to the 65 CAs.

As an alternative to the high-speed UAVs, we also conduct an additional scenario considering a common drone system with a much slower speed than the tsunami celerity^28^. Thus, the observation resembles the fixed/non-moving observation platforms. This experiment can also be used to assess the performance of moving versus non-moving observations in tsunami forecasting. Therefore, we utilize an identical number and distribution of the drones to the CAs shown in Fig. 1a. As shown in Fig. S6 (Supplementary #2), the forecast using 65 non-moving observations results in an accuracy of 83.9%, $K$ = 1.19, $\kappa$ = 1.36, which is less accurate compared to the moving observations (Fig. 3b). This clearly indicates the advantage of moving over non-moving observations. The result reconfirms the importance of the azimuthal coverage of observations in tsunami forecasting, which in this scenario is better represented by the moving than the non-moving observations. We also conduct further analyses on the effect of the number of observations to the forecast skill, which will be discussed in a later section.

### Effect of aircraft speed

The same initial aircraft distribution with various speeds results in a different level of forecast accuracy. For simplification, we assess here the influence of speed of 7 HAs (Fig. 2b) in a more general manner, thus $K$ and $\kappa$ values are omitted in the discussion. As depicted in Fig. 4a, the optimal speed to obtain the best accuracy of 76.6% is approximately 500 m/s, beyond which speed the forecast accuracy gradually decreases falling down below 70% for a speed exceeding 5000 m/s. It should be noted that the curve is based on 10-s sampling intervals (assimilation cycle). Different rates of sampling would produce different curves. In general, however, slower speeds provide more temporal than spatial information and vice versa, leading to an adverse impact on the forecasts; whereas a balanced manner can be achieved by the optimal speed due to the favorable distribution of the observation points. This is in line with our previous study^29^, concluding that observation points should be spread sufficiently wide enough to capture large-scale modes of the tsunami at which the main energy exists because a proper quantification of tsunami energy plays a critical role in tsunami forecasting^30^. The experiment also implies that extremely fast-moving platforms such as satellites (e.g., the Jason satellite orbital speed: ~7200 m/s, ground speed: ~5800 m/s) are probably not optimal. Moreover, the infrequent satellite pass-over time can be another disadvantage as well.

### Optimal number of observations and data assimilation

To determine the optimal number of observations and simultaneously asses the performance of the data assimilation procedure, we use the speed of 200 m/s similar to the CA case. We arbitrarily change the number and initial position of CAs and estimate the corresponding errors and computing times. We gradually increase the number of airplanes from 40 to 200 with an increment of 40 and plot the resulting accuracies and computing times in Fig. 4b. Additionally, the figure shows comparisons between the proposed tsunami data assimilation using a reduced rank optimal interpolation (RROI) with the standard method by an optimal interpolation (OI)^13^. Regardless of the data assimilation method, with 40 CAs, the $K$ and $\kappa$ fall beyond the suggested range^21^. This indicates that with the prescribed speed, more than 40 CAs are required to obtain the expected result. However, such an optimal number of observations by airplanes is dependent on their distribution. A relatively less number of airplanes with a better spatial distribution may produce better forecasts than more airplanes, but lacking in coverage. In terms of model performance, it is evident that the RROI can maintain a relatively short computing time, particularly for a large number of observations, compared to the OI without significantly compromising accuracy. Therefore, the RROI can efficiently cope with the moving observations, and thus meet the main requirement of a real-time tsunami forecasting.

## Discussion

Our numerical experiments suggest that the utilization of CAs as platforms for radar observations can be a promising tool for offshore large tsunami detections. However, there are still a few challenges that should be addressed in the actual implementation of the proposed approach. First is related to the technical aspect, particularly the real-time data transmission to the ground server. While the speed and bandwidth of inflight internet connections are currently limited^18^, the ADS-B system automatically broadcasts, about twice per second, information on the aircraft identification, position, altitude, speed, and other parameters obtained from onboard equipment (e.g., GNSS receiver), which can be retrieved by the ADS-B receivers installed on either terrestrial stations or on satellites^22^. For our purpose, new communication protocols or additional bandwidth to include the processed SSH data are required. Another issue that may arise is regarding cooperation with the airlines, which can be accomplished by either voluntary participation or enforced regulations by the authority. A good example is the Comprehensive Observation Network for TRace gases by AIrLiner (CONTRAIL) project^31^, in which commercial airlines provide an observational platform for obtaining free tropospheric CO_2_ worldwide^32^.

The non-technical difficulty in relation to the regulatory requirements may partly be overcome by employing HAs, commonly owned by the air force in most countries. In our study, we note that the airports indicated in Fig. 2b are selected merely to facilitate the numerical setup, such that the time required for the HAs to start detecting the tsunami is minimum as we choose only nearshore airports. In reality, some airports may not be suited for military aircraft and appropriate response time should also be taken into account. Nonetheless, this experiment shows that the limited number of platforms but with optimal speed and coverage can still be useful to enhance the forecast skill. We also demonstrate the efficacy of low-speed UAVs assuming that continuous tsunami monitoring is available. Considering that the total weight and dimension of the required devices (GNSS receiver, radar altimeter^33^, and ADS-B transponder) are relatively small (https://www.garmin.com, accessed Dec. 2019), it is practically possible to install it on small-sized aircraft.

In this study, the success of the tsunami data assimilation is to some extent attributed to the estimate of tsunami source considered as the initial guess for the background state. The source was inferred from the virtually observed tsunami at the DONET system and GPS buoys^17^. However, similar estimates can be difficult to obtain at regions with a limited number of tsunami observation stations. To resolve this issue, Sheehan *et al*.^15^ proposed the use of a rapid W-phase earthquake solution to initiate the tsunami data assimilation. The approach is feasible as seismic instrumentations are more globally available than that of the tsunami. The tsunami source can also be estimated from onshore GNSS data in real-time^11^. Initially, the tsunami data assimilation was developed to produce tsunami forecasts without the need to account for the tsunami source^13^ that is often accompanied by a higher degree of uncertainty^34^. However, the lack of spatial density of observation points greatly degrades the forecast performance of the method^35^. Similarly, in our study, the distance between observation platforms can be very far at a certain instant in time. Therefore, the initial guess is necessary to minimize artificial shocks caused by the data assimilation procedure normally appeared in the early stages^36^. The combined or individual use of seismic, geodetic, and tsunami data should be considered in the tsunami source estimation to ensure the universality of the method.

Similar to the ship height observations^9,10^, a unique and important feature of the proposed observational scheme lies in the utilization of existing platforms that are expected to provide more sustainable observations in the future as the number of CAs are continuously growing over time. Furthermore, it may also lead to a relatively cost-effective observation as the required devices are available in the present avionic system. However, we note that necessary customizations are needed and the proposed system is likely subjected to several obstacles during the actual operation. The airborne observations regardless of the platforms are prone to extreme weather conditions, and the operation of commercial drones should comply with the local regulations mainly for safety purposes. Furthermore, the terrestrial ADS-B receiver can also be interfered with or damaged by the preceding earthquake. Nevertheless, combined with the existing tsunami observing systems, we would enhance the tsunami warning capability.

Millions of people around the world and US$ billions in assets are exposed to the global tsunami hazard^37^. Such a problem requires a comprehensive solution, in which tsunami monitoring and forecasting play an important role; hence, exploring innovative ways is of necessity. A recent initiative utilizing undersea telecommunication cables for global ocean observations called Science Monitoring And Reliable Telecommunications (SMART) has been introduced^38^. Howe *et al*.^38^ demonstrated the effectiveness of the SMART system using hypothetical cable routes to improve the warning system of the Pacific Tsunami Warning Center for a far-field event. Our study is focused on a near field application, although the results may not fully address the issue of timeliness particularly at the shorelines adjacent to the source region, for the tsunami may arrive earlier than 10 min. In this case, one solution is to raise community awareness, as noted by Kânoğlu *et al*.^39^ and Okal^40^, in which statistics from several recent tsunami events suggested that self-evacuation by educated population, presently remains the most effective way for the near field tsunami mitigation. Apart from the near field and local application to Japan, the study can be seminal to broader implications. With the vast coverage of unconventional platforms such as CAs, the new system can also lead to global tsunami monitoring. Although the developments of tsunami observing system introduced in this study are still in its infancy, it could potentially be a viable future technology, not only limited to natural disaster-related applications. A global monitoring of sea surface states will also be valuable to other variety of marine operations (e.g., fisheries, coastal development planning, marine renewable energy, etc.). Since the system is mutually beneficial to both disaster and commercial aspects, similar to the SMART project, the joint use of the data may be capitalized to ease the financial burden concerning the operational costs. Lastly, the rapid advancements of aviation, observation, and information technology, as well as the supporting legislation, are of importance to expedite the realization of the proposed approach.

## Method

### Data acquisition

**Figure 5:**
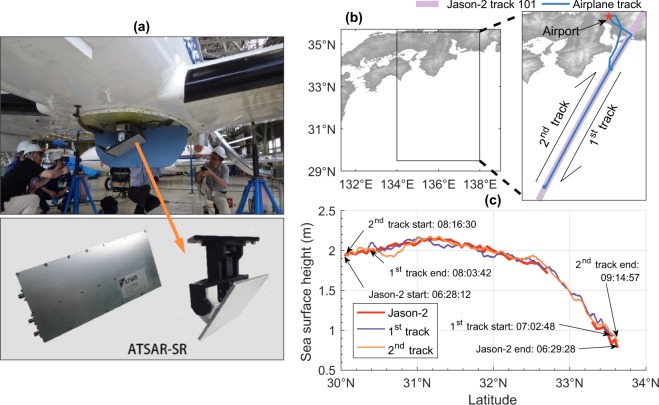
SSH measurements on 29 June 2016. (**a**) Alouette Technology Synthetic Aperture Radar (ATSAR) hardware installed on an airplane. (**b**) Airplane tracks overlaid by the Jason-2 track. (**c**) Comparison of SSH observed by the ATSAR and Jason-2. Time annotation is based on the global time zone (UTC). Images in (**a)** were taken by authors and available from http://www.altek.jp/. Maps in (**b**) are produced by authors based on GEBCO_08 Grid data (https://www.gebco.net/), using MATLAB 2016b.

We have conducted airborne measurements of SSH using an airplane equipped with a nadir-pointing frequency-modulated continuous wave radar and a GNSS receiver. In the real implementation, however, an additional device is required for real-time data transmissions that are either ADS-B transponder or onboard modem. The SSH can be obtained by subtracting the distance between the airplane and ocean surface measured by the radar from the absolute altitude of the airplane relative to a reference of Earth ellipsoid retrieved by the GNSS positioning method. The summary of the observation procedure is presented in Supplementary #1 and Fig. S2. While details about the data processing, observation parameters, radar specifications, as well as more results of field observations can be found in Hirobe *et al*.^18^. Figure 5 shows examples of the result of the radar observations conducted on 29 June 2016. We validate the results via a comparison with the Jason-2 SSH. Since the accuracy of the Jason-2 SSH is very high (<3.5 cm)^41^, we assume that the noise level of our measurements is simply equivalent to the residual between the observed SSH and the Jason-2 SSH. The noise characterization result with an estimated observation error of ~10 cm is then incorporated into the data assimilation procedure. A sample of virtually observed tsunami elevation used in the data assimilation is shown in Fig. S7.

Figure 5c depicts the variation of SSH along the airplane tracks (Fig. 5b) after removal of the tide and the equipotential surface of the Earth’s gravity field known as the geoid. The curve and approximately 1 m SSH difference at both ends of the track are associated with the Kuroshio current. Such an SSH variation exhibits almost a similar spatial scale to large tsunamis typically generated in the megathrust region such as our study area, the Nankai Trough of Japan. Therefore, it is reasonable to regard the Kuroshio current as a proxy for the tsunami. Although in the actual application, it is necessary to isolate the tsunami signal from other oceanic phenomena including the Kuroshio current, mesoscale eddies, and tide to facilitate the tsunami forecasting algorithms. This tsunami extraction feature should be incorporated in the radar data processing stages^18^.

### Tsunami data assimilation

The standard tsunami data assimilation procedure is based on a sequential OI^13^. The gain matrix in the OI is assumed to be constant because it depends only on observations-to-grids and observations-to-observations distances expressed by a Gaussian kernel (we use an e-folding scale of 23 km). This assumption significantly simplifies the computation, which is crucial for tsunami forecasting purposes. Despite such a rough assumption, previous studies^14–16^ proved that the method is sufficient for a transient process such as tsunamis using real case data. However, for moving observation platforms, the assumption is no longer valid since the relative distances from observation points to both other observation points and the grids would change with time. Consequently, the gain matrix must be computed at each assimilation cycle, which inevitably increases the computing time, particularly when a considerable number of observations are taken into account. We, therefore, apply a rank reduction for the background error covariance matrix called the RROI. Similar rank reduction in various data assimilation schemes has previously been introduced e.g.^42–44^.

Following the rank reduction approach in Song & Lim^44^, the $n\times n$ background error covariance ${\bf{B}}$, where $n$ is the number of numerical model grids, can be decomposed into orthonormal eigenvectors ${{\boldsymbol{e}}}_{j}$ with the corresponding eigenvalues ${\lambda }_{j}$, that is

1 $${\bf{B}}=\mathop{\sum }\limits_{j=1}^{n}{\lambda }_{j}{{\boldsymbol{e}}}_{j}{{\boldsymbol{e}}}_{j}^{T}.$$

The ${\bf{B}}$ matrix does not depend on measurements, so it can be computed and decomposed in advance, and stored. We then select only *r* leading components of the eigenvalues and eigenvectors based on a trial-and-error approach. Figure S8 shows that the curve of model error remains relatively flat at >70 eigenmodes (*r*). With the same level of error, the computing time escalates to more than 0.5 min when *r* > 110. Therefore, we use *r* = 110 ($\ll n$), signifying the reduced-rank approach. From this decomposition, we can construct an $r\times r$ diagonal matrix ${\bf{D}}$,

2 $${\bf{D}}=[\begin{array}{ccc}{\lambda }_{1} & \cdots  & 0\\ \vdots  & \ddots  & \vdots \\ 0 & \cdots  & {\lambda }_{r}\end{array}],$$

and an $n\times r$ leading eigenvector matrix ${\bf{E}}$,

3 $${\bf{E}}=[{{\boldsymbol{e}}}_{1}\,{{\boldsymbol{e}}}_{2}\,\ldots \,{{\boldsymbol{e}}}_{r}].$$

The $n$ background state vector ${{\boldsymbol{x}}}^{b}\,$can be represented using a reduced rank approximation by transforming it into *r*-dimensional state vector ${{\boldsymbol{x}}}^{bl}$ as,

4 $${{\boldsymbol{x}}}^{bl}={{\bf{E}}}^{T}{{\boldsymbol{x}}}^{b},$$

while the trailing eigenmodes for the background state is formulated as,

5 $${{\boldsymbol{x}}}^{bt}={{\boldsymbol{x}}}^{b}-{\bf{E}}{{\boldsymbol{x}}}^{bl}.$$

If the number of observations is *p*, the $r\times p$ gain matrix in the reduced rank expression can be written as,

6 $${{\bf{W}}}^{l}={({{\bf{D}}}^{-1}+{[{\bf{HE}}]}^{T}{{\bf{R}}}^{-1}{\bf{HE}})}^{-1}{({\bf{HE}})}^{T}{{\bf{R}}}^{-1},$$

where ${\bf{H}}$ is the observational operator matrix ($p\times n$) and ${\bf{R}}$ is the error covariance matrix of the observations ($p\times p$). We assume that ${\bf{R}}$ is a diagonal matrix, in which its diagonal component consists of normalized observation error variances ($\rho$)^45^. In this study, we set $\rho$ = 0.9 reflecting the noise level in the real observation. The *r*-dimensional reduced rank analysis state vector is then written as,

7 $${{\boldsymbol{x}}}^{al}={{\boldsymbol{x}}}^{bl}+{{\bf{W}}}^{l}({\boldsymbol{y}}-{{\bf{H}}}^{l}{{\boldsymbol{x}}}^{bl}),$$

where the observation operator ${{\bf{H}}}^{l}{{\boldsymbol{x}}}^{bl}={\bf{H}}({\bf{E}}{{\boldsymbol{x}}}^{bl}+{{\boldsymbol{x}}}^{bt})$ and ${\boldsymbol{y}}$ is a vector of observed tsunami elevations with a dimension of *p*. The following equation is then used to transform the reduced rank analysis state into a full rank *n*-dimensional space,

8 $${{\boldsymbol{x}}}^{a}={\bf{E}}{{\boldsymbol{x}}}^{al}+{{\boldsymbol{x}}}^{bt}.$$

In this sequential assimilation, the background state vector at the *i*-th time step is updated by integrating the shallow water equations^46^ using the analysis state vector at the previous time step (*i*-1), i.e. ${{\boldsymbol{x}}}_{i}^{b}\equiv {\bf{F}}{{\boldsymbol{x}}}_{i-1}^{a}$. The bathymetry data are based on the GEBCO_08 Grid (30 arc sec). We set the assimilation cycle at every 10 s, the forward model time step of 1 s, and 10-min assimilation window of 60-min simulation time. Note that we only use tsunami elevation data for the assimilation, regarding the velocity components as unobserved variables. We apply a simplified cross-covariance to statistically spread the information from observations among variables and an initial guess for the background state similar to Mulia *et al*.^17^.

## Supplementary information

Supplementary information.

## Data Availability

The airborne observed-SSH data are stored in 10.5281/zenodo.1409374. The satellite Jason-2 SSH data are provided by the NASA EOSDIS Physical Oceanography Distributed Active Archive Center at the Jet Propulsion Laboratory (https://podaac.jpl.nasa.gov/dataset/OSTM_L2_OST_OGDR_GPS). The bathymetry data are available at https://www.gebco.net/. The CAs locations are based on a real-time airplanes distribution obtained from https://www.flightradar24.com.
