# Supplementary material for: Advanced tsunami detection and forecasting by radar on unconventional airborne observing platforms
Source: Sci Rep. 2020 Feb 12;10:2412. doi: 10.1038/s41598-020-59239-1 (PMC7016180; doi:10.1038/s41598-020-59239-1)
Supplement: Supplementary file 1 — Supplementary information. [file 41598_2020_59239_MOESM1_ESM.pdf]

Supplementary Information for

Advanced tsunami detection and forecasting by radar on unconventional airborne observing platforms

Iyan E. Mulia[1,2*], Tomoyuki Hirobe[1,3], Daisuke Inazu[4], Takahiro Endoh[5], Yoshihiro Niwa[1,6], Aditya Riadi Gusman[7], Hidee Tatehata[1,3], Takuji Waseda[1,8], and Toshiyuki Hibiya[1,9]

[1]UTokyo Ocean Alliance, The University of Tokyo, Tokyo, Japan.
[2]Earthquake Research Institute, The University of Tokyo, Tokyo, Japan.
[3]Japan Weather Association, Tokyo, Japan
[4]Department of Marine Resources and Energy, Tokyo University of Marine Science and Technology, Tokyo, Japan.
[5]Research Institute for Applied Mechanics, Kyushu University, Fukuoka, Japan.
[6]Center for Ocean Literacy and Education, The University of Tokyo, Tokyo, Japan
[7]GNS Science, Lower Hutt, New Zealand.
[8]Graduate School of Frontier Sciences, The University of Tokyo, Chiba, Japan.
[9]Graduate School of Science, The University of Tokyo, Tokyo, Japan.

[*]iyan@eri.u-tokyo.ac.jp.

Supplementary #1: Experiment procedures, statistical evaluations, summary of airborne radar observation

S1. 1. Experimental setup

The overall step-by-step procedures of the twin-data experiment in our study depicted in Fig. S.1 are as follows:

1. Perform the tsunami simulation based on the shallow water equation using the hypothetical tsunami source for 60-min simulation time. We regard the simulated tsunami wavefield as the true state to be approximated.

2. Extract the tsunami elevation along the assumed airplane tracks for the data assimilation (10-min observations).

3. Extract the maximum coastal tsunami heights for model validation purposes (Fig. 1e in the main text).

4. Add the noise from real observation to the synthetic tsunami elevations obtained in Step 2. We then consider the noisy true states as pseudo-observations.

5. Perform the RROI by assimilating the pseudo-observations in Step 4 for assimilation period of 10 min and assimilation cycle of 10 s. A rough initial

estimate for the background state is obtained from an inversion analysis (*Mulia et al*., 2017).

6. Validate the RROI result through the comparison with the true state from Step 3.

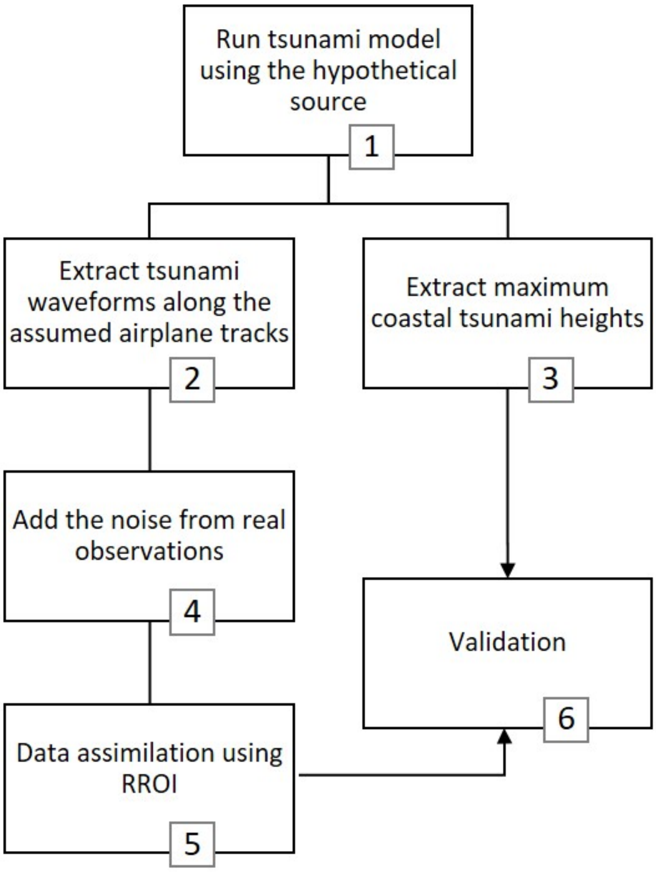

Figure S.1. Flow diagram of the experimental setup.

S1. 2. Statistical evaluations

To assess the forecast performance, we compare the maximum coastal tsunami heights from the true state (observed) with the assimilation result (modeled). We use statistical measures based on geometric mean ratio (K) of observed (O) to modeled (M) at point i, together with its standard deviation κ (*Aida*, 1978) as,

$$\log K = \frac{1}{N}\sum_{i=1}^{N} \log \frac{O_i}{M_i}, \qquad \text{(s1)}$$

$$\log \kappa = \sqrt{\frac{1}{N}\sum_{i=1}^{N}\left(\log \frac{O_i}{M_i}\right)^2 - (\log K)^2}. \quad \text{(s2)}$$

Shuto (1991) suggested values of 0.8 < K < 1.2 and κ < 1.4 as criteria for satisfactory results. In addition to equations (s1) and (s2), we also include quantification of accuracy in percentage as follows,

$$\text{Accuracy}(\%) = \begin{cases} 1/K \times 100, & K \geq 1 \\ K \times 100, & K < 1 \end{cases}. \quad \text{(s3)}$$

S1. 3. Summary of airborne radar observation

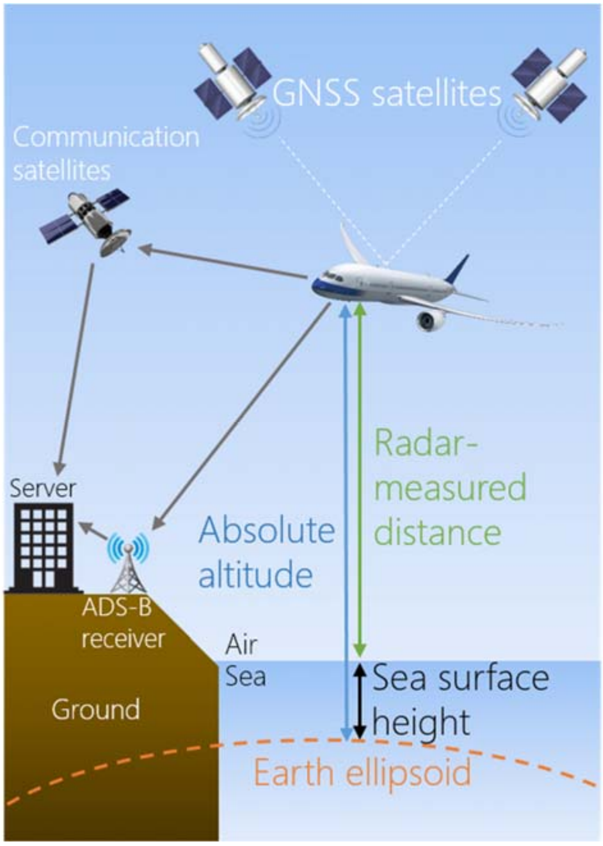

Figure S.2. Schematic of the airborne radar observation. Figure is created by authors using INKSCAPE 0.92 software (https://inkscape.org/), and images are available from https://freepngimg.com/.

The proposed airborne radar observation shown in Fig. S.2 can be summarized as follows:

1. The radar transmitter emits electromagnetic signals in the nadir direction, then the reflected signals by the sea surface are retrieved back by the radar receiver. The distance from the radar to the sea surface can then be calculated from the known signal properties, such as frequency, bandwidth, and sweep time (*Hirobe et al.*, 2019).

2. Concurrently, a Global Navigation Satellite System (GNSS) receiver pick up signals from constellations of satellites that provides information on the absolute altitude relative to a reference of Earth ellipsoid. We apply a positioning method based on a real-time kinematic (RTK) technique.

3. The sea surface height is obtained by subtracting the radar-measured distance (point 1) from the absolute altitude resulted by the GNSS analysis (point 2).

4. The data is automatically broadcasted through the automatic dependent surveillance-broadcast (ADS-B) system or transferred via satellite internet connections. The ADS-B receiver can be either terrestrial or satellite-based station.

References

Aida, I. Reliability of a tsunami source model derived from fault parameters. *Journal of Physics of the Earth*, **26**(1), 57-73 (1978).

Hirobe, T. et al. Observation of sea surface height using airborne radar altimetry: a new approach for large offshore tsunami detection. *Journal of Oceanography*, **75**, 541-558 (2019).

Mulia, I. E., Inazu, D., Waseda, T., and Gusman, A. R. Preparing for the future Nankai Trough tsunami: A data assimilation and inversion analysis from various observational systems. *Journal of Geophysical Research: Oceans*, **122**(10), 7924-7937 (2017).

Shuto, N. Numerical simulation of tsunamis – its present and near future. *Natural Hazard*, **4**, 171 – 191 (1991).

Supplementary #2: Figures of additional forecast scenarios and sensitively analysis

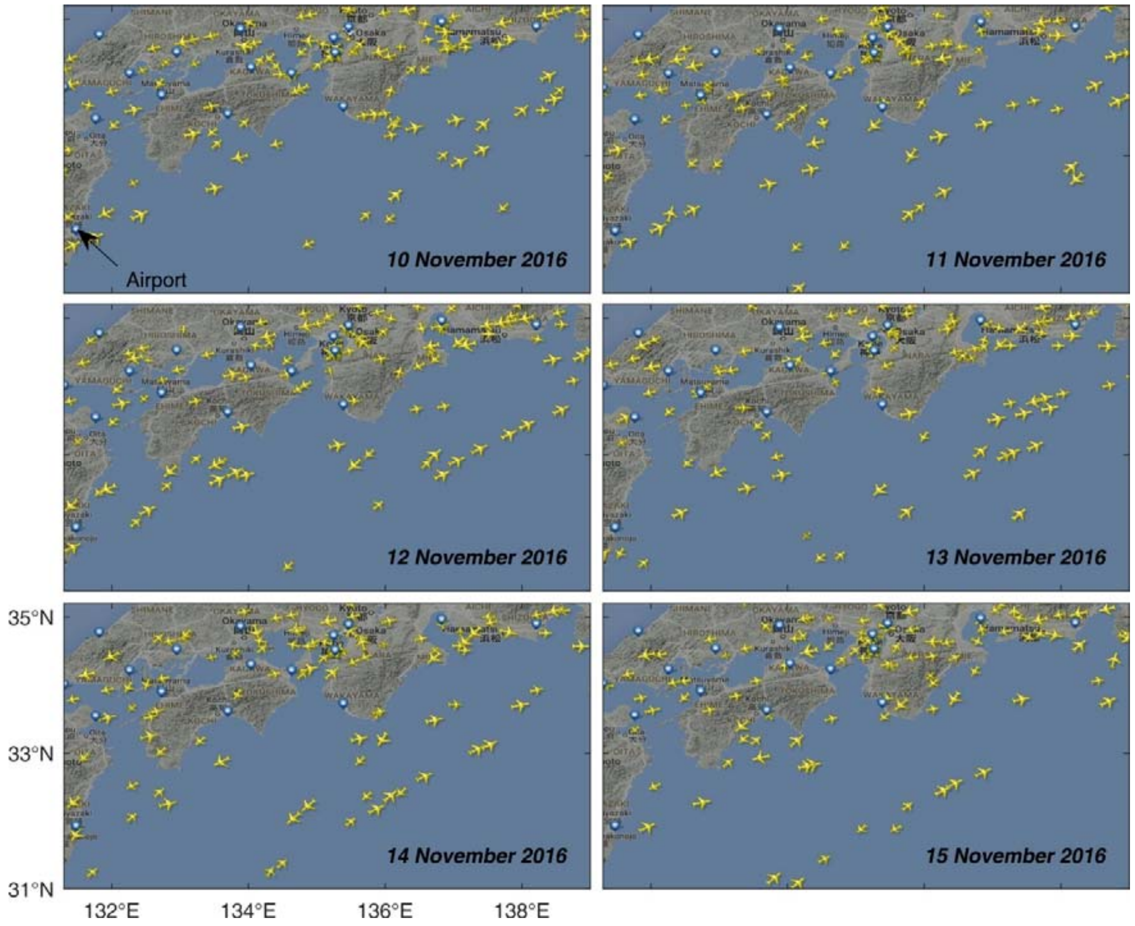

Figure S.3. Commercial airplane (CA) distributions for six consecutive days around the study area at 05:00 UTC obtained from www.flightradar24.com. We use the 15 November 2016 data as a sample to test the proposed method. Images are obtained from https://www.flightradar24.com/ (data: Google Maps, 2016, flightradar24) modified by authors using MATLAB 2016b.

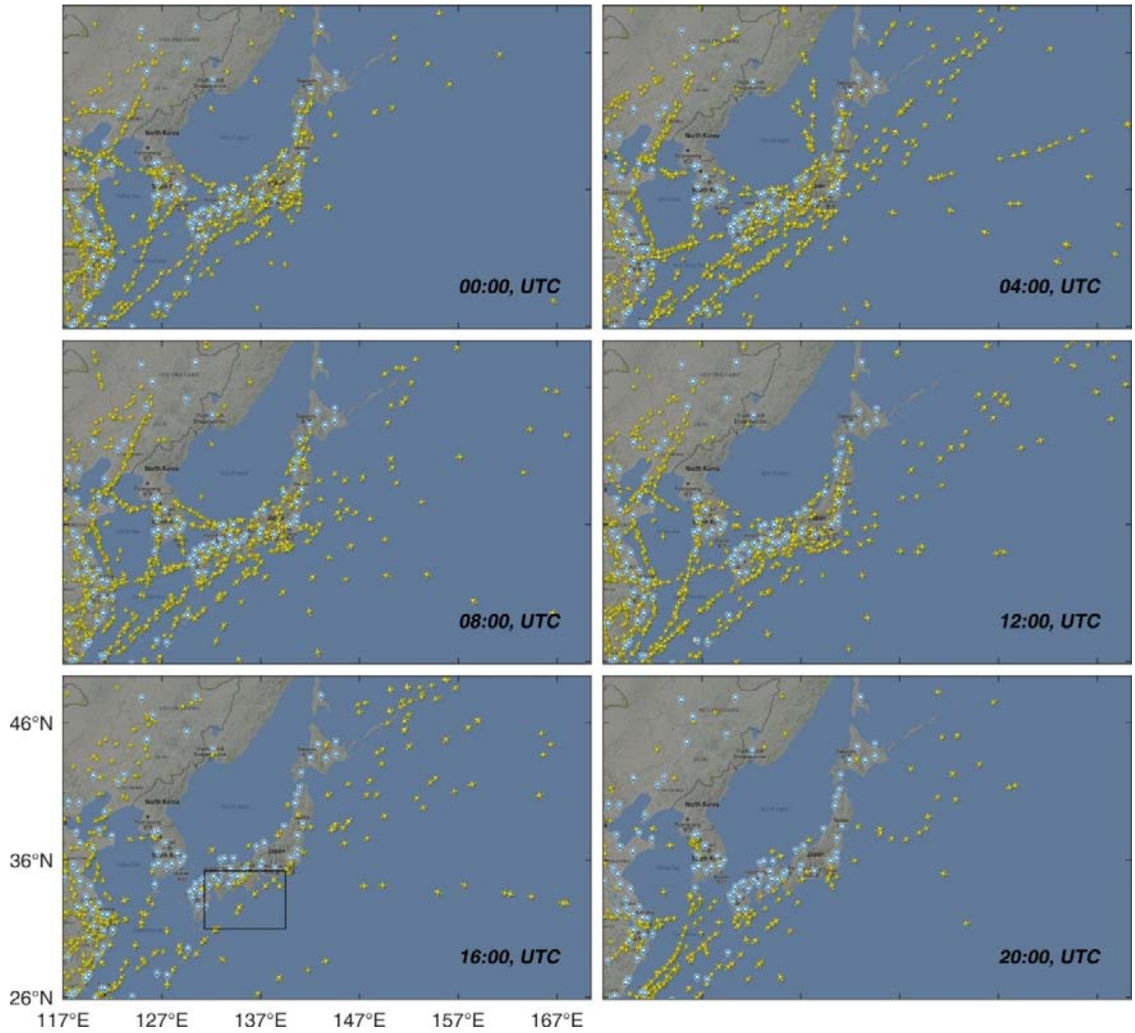

Figure S.4. CA distributions throughout Japan at 4-hour intervals on 25 July 2018 obtained from www.flightradar24.com. We also apply the proposed method to the 16:00 UTC (+9 JST) data, which exhibit the smallest number of CAs in the study area (black rectangle) among all the snapshots. Images are obtained from https://www.flightradar24.com/ (data: Google Maps, 2016, flightradar24) modified by authors using MATLAB 2016b.

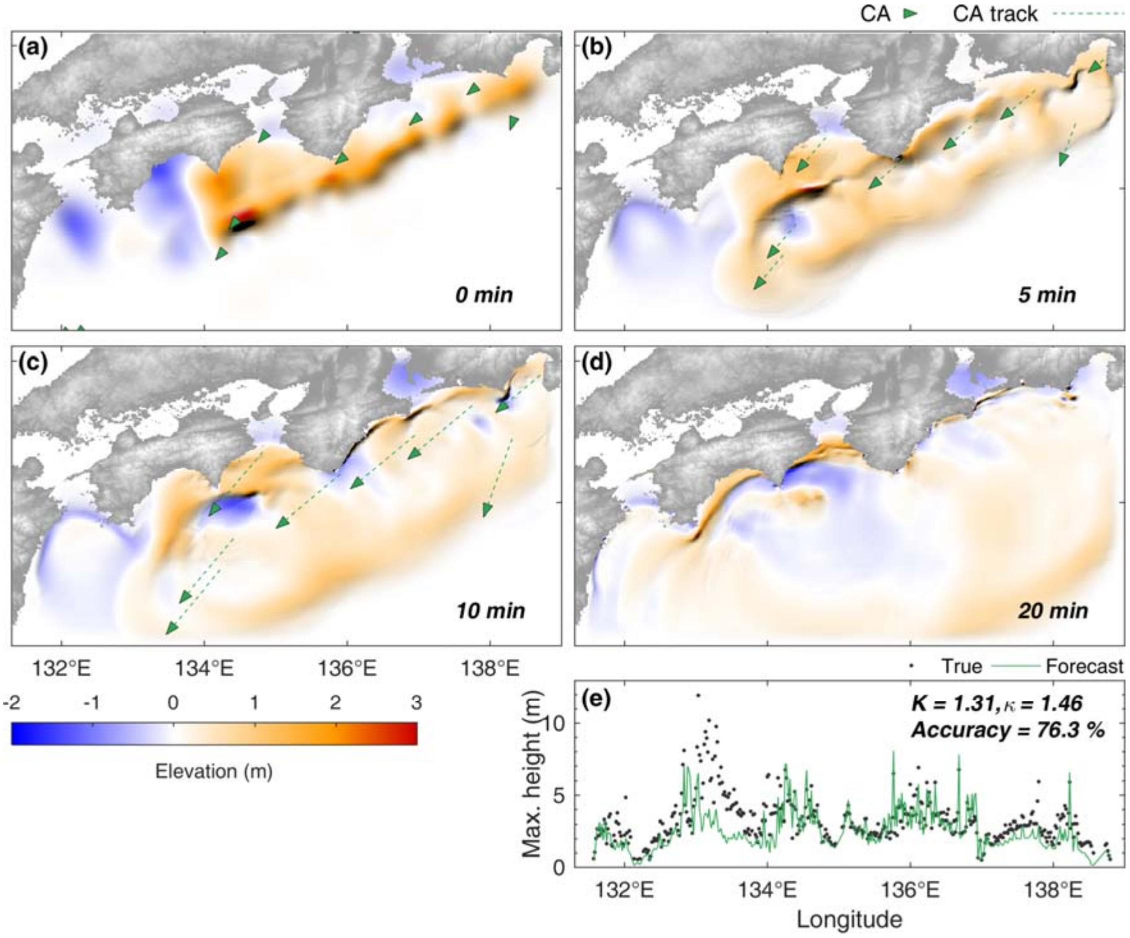

Figure S.5. (a)-(d) Assimilated tsunami elevations using 8 CAs with 10-min assimilation period based on the 25 July 2018 data (16:00 UTC). The initial condition (at t = 0) is obtained from the inversion analysis. (e) Comparison of true and forecasted maximum coastal tsunami heights obtained after 60-min simulation. Maps in (a-d) are produced by authors based on GEBCO_08 Grid data (https://www.gebco.net/), using MATLAB 2016b.

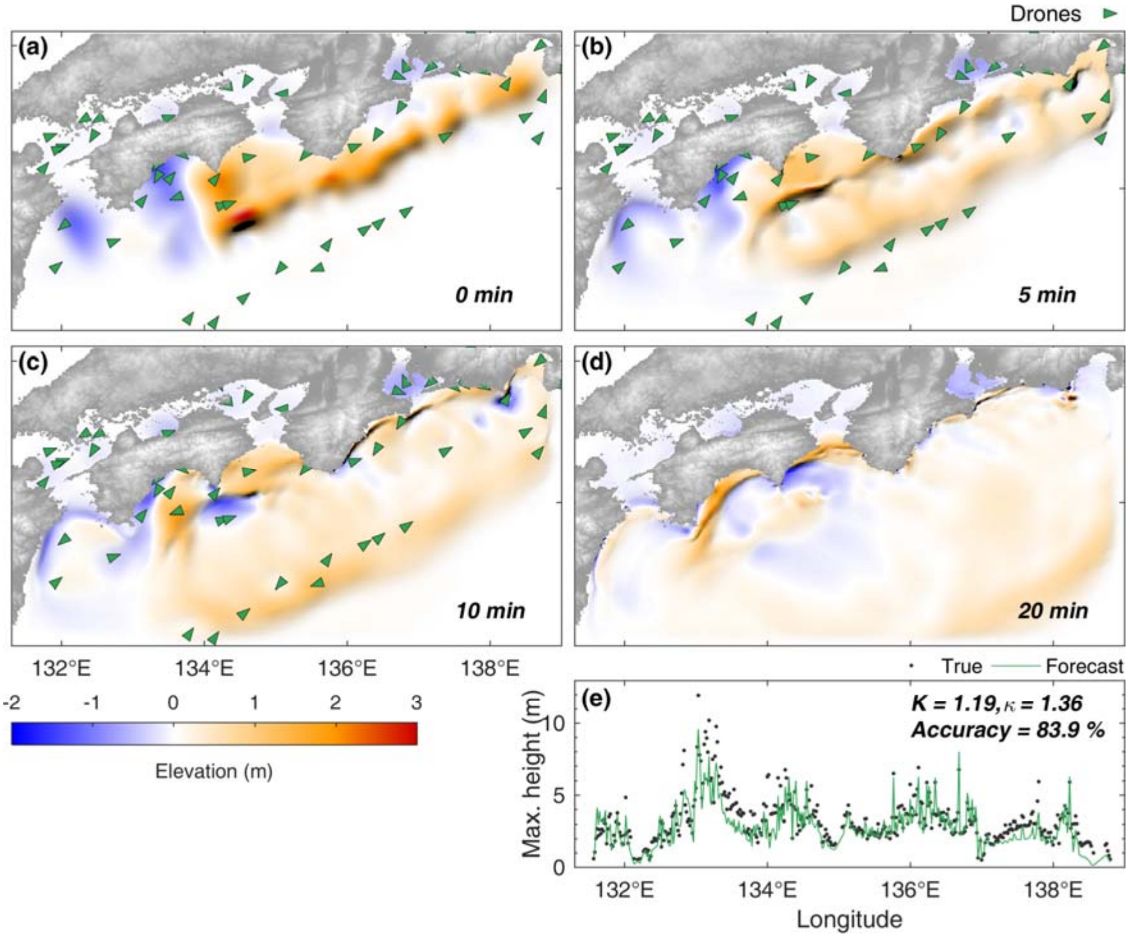

Figure S.6. (a)-(d) Assimilated tsunami elevations using 65 slow-speed drones assumed as fixed/non-moving observation platforms, with 10-min assimilation period. The initial condition (at t = 0) is obtained from the inversion analysis. (e) Comparison of true and forecasted maximum coastal tsunami heights obtained after 60-min simulation. Maps in (a-d) are produced by authors based on GEBCO_08 Grid data (https://www.gebco.net/), using MATLAB 2016b.

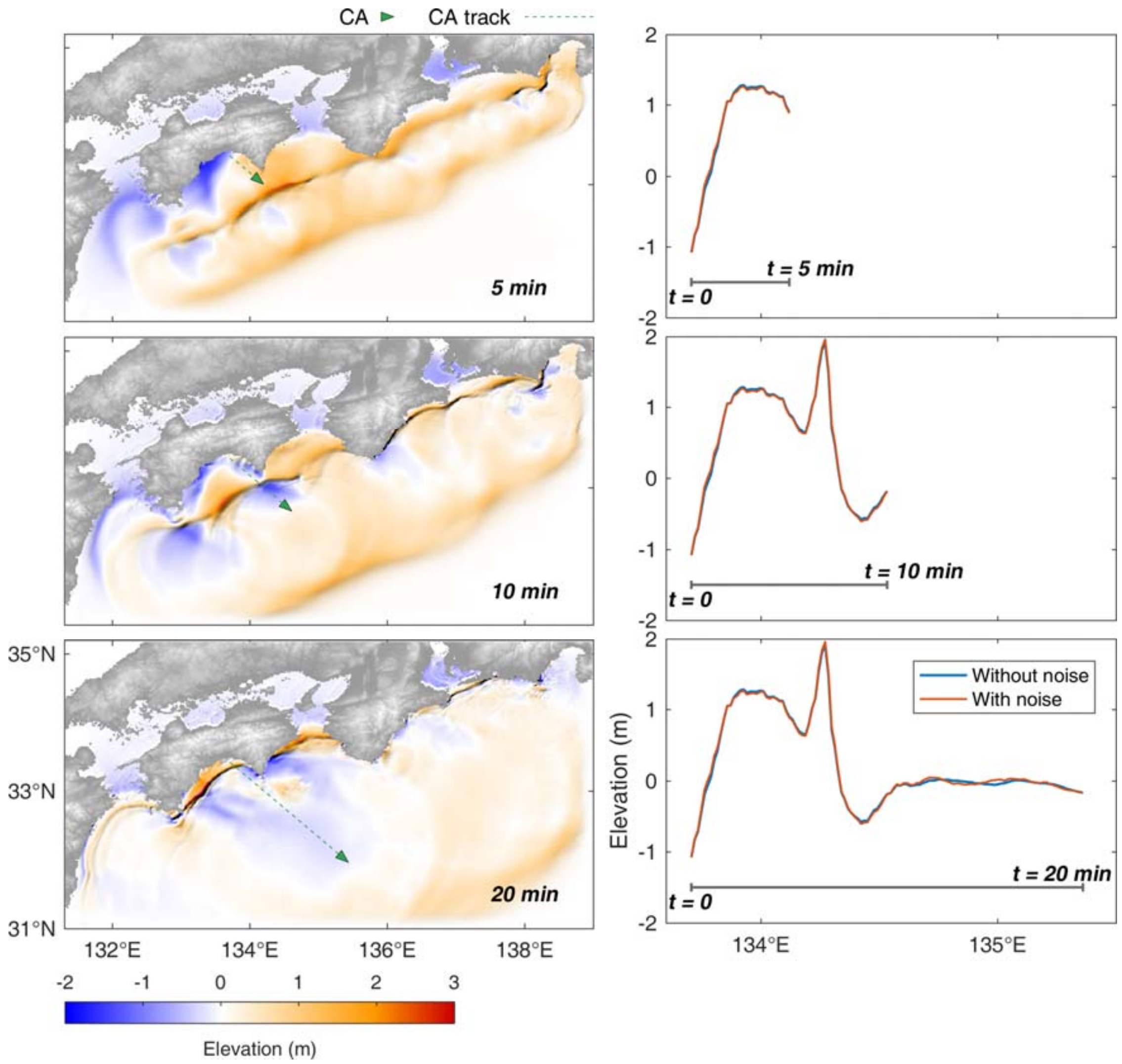

Figure S.7. Virtually observed tsunami by an airplane. Left panel shows the true state of tsunami elevation overlaid with airplane tracks at 5, 10, and 20 min. Right panel depicts the corresponding observed spatiotemporal variations of tsunami elevations along the tracks. The noise is obtained from our observation (see method section in the main text). Maps are produced by authors based on GEBCO_08 Grid data (https://www.gebco.net/), using MATLAB 2016b.

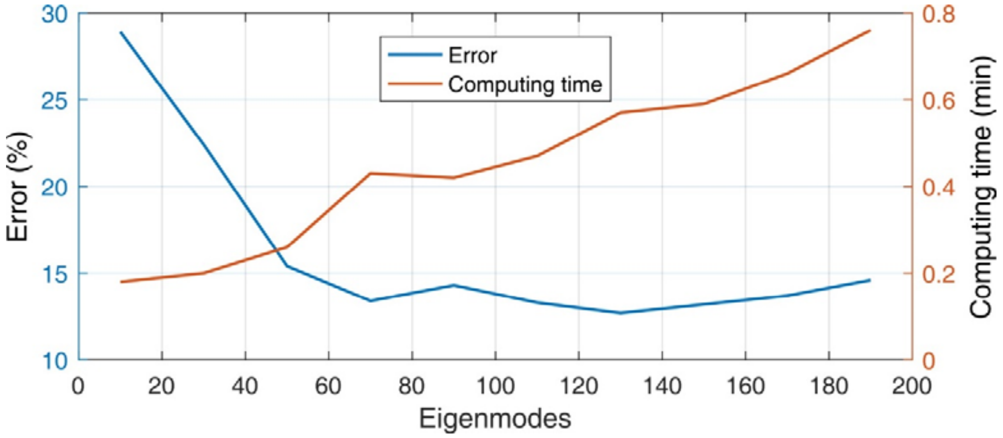

Figure S.8. Errors (100 % - accuracy (equation s3)) and computing times based on the considered number of eigenmodes ($\boldsymbol{r}$) in the RROI using 65 airplanes with 10-min assimilation period.
